# Clinicopathological characteristics and prognostic analysis of tumor-infiltrating lymphocytes (TILs) in ductal carcinoma in situ (DCIS) and DCIS with microinvasion (DCIS-Mi) of the breast

**DOI:** 10.1007/s10549-022-06553-z

**Published:** 2022-03-08

**Authors:** Huiqing Jia, Peng Zhao, Zhaoxu Chen, Guanqun Wang, Xianning Dong, Xiaoming Xing, Xiaohua Tan, Chengqin Wang

**Affiliations:** 1 Department of Pathology, the Affiliated Hospital of Qingdao University, Qingdao, 266000 Shandong China; 2 Department of Pathology, School of Basic Medicine, Qingdao University, Qingdao, 266021 Shandong China

**Keywords:** TILs, DCIS, DCIS-Mi, HER2+, DFS

## Abstract

**Objective:**

Our purpose is to evaluate the correlation of TILs with clinicopathological characteristics and disease free survival (DFS) in DCIS and DCIS-Mi breast cancer (BC) patients.

**Methods:**

We retrospectively reviewed the data of 360 DCIS patients and 125 DCIS-Mi patients treated by a single institution from 2016 to 2019. TILs are regarded as continuous variables and are divided into low (≤ 5%), medium (5–40%) and high (≥ 40%) for statistical analysis.

**Results:**

In DCIS and DCIS-Mi patients, larger tumor size, higher nuclear grade, hormone receptor (HR) negativity and human epidermal growth factor receptor 2(HER2) overexpression are all related to high TILs (*P* < 0.05). In addition, compared with DCIS, DCIS-Mi patients were significantly associated with high TILs (*P* < 0.001). Based on the different results of the subtypes, we further studied the correlation between TILs and DFS in 279 cases of HER2+ patients (204 of DCIS; 75 of DCIS-Mi). In HER2+ group, DCIS-Mi was significantly associated with HR negativity (*P* = 0.015) and high TILs (*P* = 0.002) compared with DCIS patients. In the survival analysis, we found that TILs had no effect on the DFS of DCIS (*P* = 0.938), DCIS-Mi (*P* = 0.807), and HER2+ (*P* = 0.379) BC patients. In the univariate and multivariate cox regression analysis, the correlation between TILs and the prognosis of DFS has not been confirmed in the three BC groups (*P* > 0.05).

**Conclusion:**

TILs have played an non-negligible role in the progress of DCIS to DCIS-Mi, especially in HER2+ BC. The predictive and prognostic value of TILs still needs further research to confirm.

**Supplementary Information:**

The online version contains supplementary material available at 10.1007/s10549-022-06553-z.

## Introduction

With the increase of public awareness of BC screening and the widespread use of mammograms, the detection rate of DCIS has greatly increased, accounting for about 20–25% of BC [1]. Among DCIS lesions, micro-invasive lesions (≤ 1 mm) can be found in about 5–10% of DCIS, which we call DCIS-Mi. In general, approximately 50% of invasive breast cancer (IBC) have progressed from DCIS [2]. DCIS-Mi is generally considered to be the transitional stage of DCIS developing into an aggressive disease [3]. In recent years, the immunotherapy of BC has become a promising treatment method, which has triggered in-depth research on the tumor microenvironment (TME)[4]. As an important part of TME, TILs mainly include T cells, B cells, and natural killer (NK) cells. Among them, T cells dominate adaptive immunity is the key to effective and sustained anti-tumor response. TILs have been described in many solid tumors including BC. Furthermore, stromal TILs have been proven to be a valuable and independent prognostic indicator in triple-negative breast cancer (TNBC) [5]. In TNBC and HER2+ patients receiving neoadjuvant chemotherapy (NAC), high density TILs are associated with higher pathologic complete response (pCR) rate and better survival benefits [6]. Immune cell infiltration of tumor is usually an early event of BC. Relevant studies have shown that TILs have an effect on the local destruction of myoepithelial cells associated with tumor invasion in the early stage [7]. The number and function of TILs may have changed during the infiltration of DCIS. However, research on TILs in BC pre-invasive lesions is still limited, and we have insufficient information on the biological behavior and survival prognosis of DCIS and DCIS-Mi.

Therefore, this study is to evaluate the density of stromal TILs in DCIS and DCIS-Mi BC patients, and further analyze its correlation with the clinicopathological characteristics and prognosis of BC patients, in order to try to find out the potential prediction or prognosis markers of DCIS.

## Materials and methods

### Patients and clinicopathological data

In this retrospective study, we included 485 patients with primary BC diagnosed in the Affiliated Hospital of Qingdao University between 2016 and 2019, including 360 cases of DCIS and 125 cases of DCIS-Mi. All cases of DCIS-Mi were reviewed by a senior pathologist (CQ.W).Patients with simultaneous bilateral breast cancer (interval between diagnosis of tumors on both sides < 6 months) and receiving NAC were excluded. Clinicopathological information (including age, tumor size, nuclear grade, ki67 index, HR status, HER2 status, surgical operation) were collected through clinical medical records and pathology databases. DFS is defined as the time interval from surgery of BC patients to disease progression (including ipsilateral or contralateral recurrence of BC, local/distant metastasis) or death. The follow-up ended in November 2021.

### Pathology methods

According to the AJCC/WHO standard [8, 9], DCIS is an epithelial hyperplastic disease which is confined within the basement membrane of the mammary ductal–lobular system. We define DCIS-Mi BC as the main tumor of the DCIS lesion, where the tumor cells break through the basement membrane and spread to the interstitial tissue, and the infiltrating lesion ≤ 1 mm. HER2+ is defined as immunohistochemistry (IHC) score of 3 + , or fluorescence in situ hybridization (FISH) to detect gene amplification. According to the expression status of HR and HER2, molecular subtypes are divided into: HR + HER2−; HR + HER2+; HR-HER2+ and TNBC. HR positive is divided into ER + PR + , ER + PR− and ER− PR + . ER, PR and Ki-67 positive are all defined as ≥ 1% of tumor cells with nuclear staining assessed by IHC. When Ki-67 > 20% of tumor cells show nuclear staining, the proliferation index is high.

### TILs assessment in DCIS and DCIS-Mi patients

**Fig. 1:**
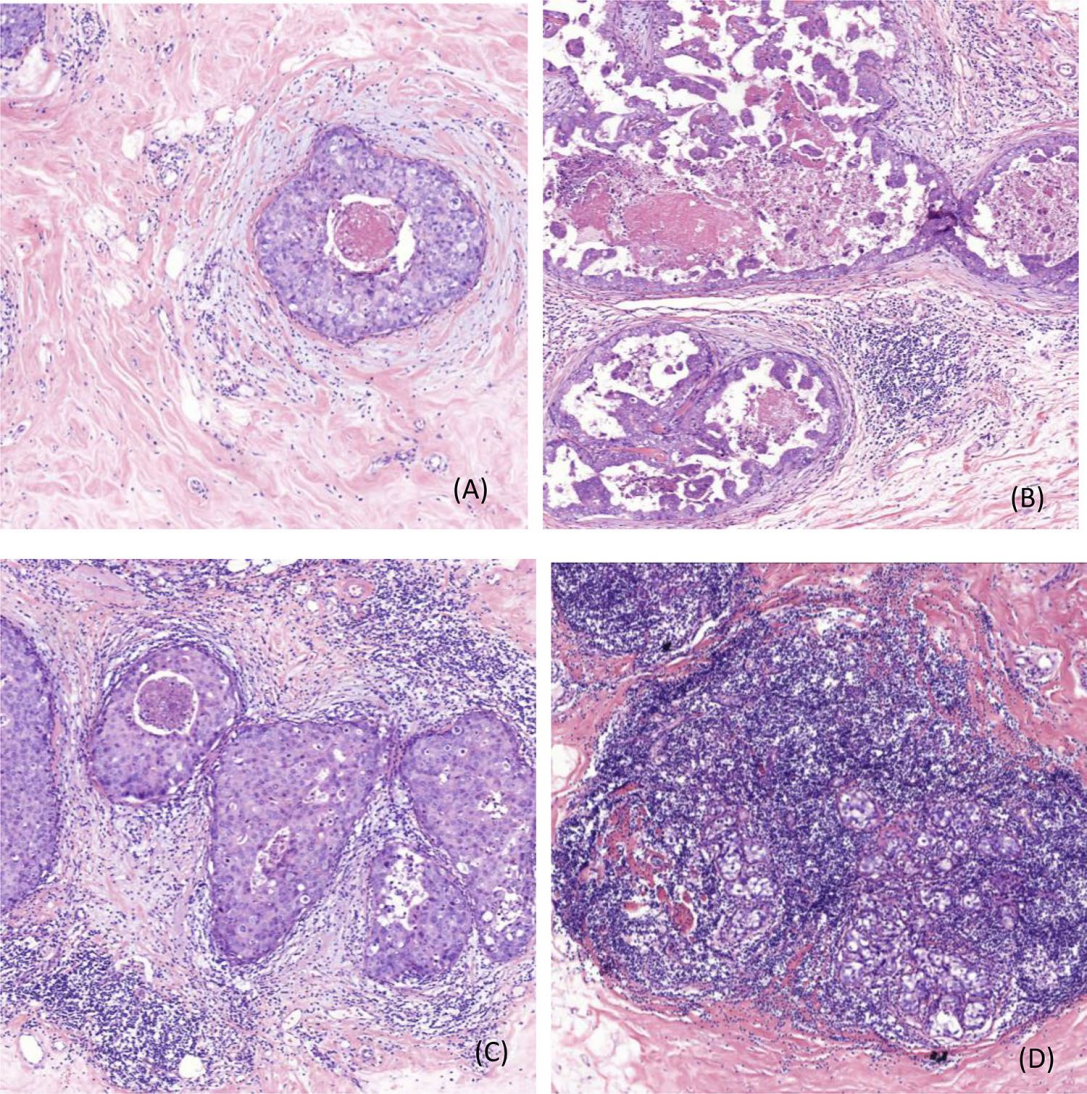
H&E images of TILs density in ductal carcinoma in situ: **A:** TILs < 5%; **B:** TILs = 10%; **C:** TILs = 40%; **D:** TILs > 90% (× 100 magnification)

There is no uniform evaluation guideline for stromal TILs in DCIS. According to the recommendations of the International Immuno-Oncology Biomarkers Working Group [10], we adopted the method of Pruneri et al. [5]. Stromal TILs were assessed as the ratio of the area occupied by mononuclear inflammatory cells to the total intratumoral stromal area. All mononuclear cells (including lymphocytes and plasma cells) should be scored, but polymorphonuclear leukocytes are excluded. The stromal area was defined as the specialized stroma surrounding the ducts involved in carcinoma in situ, or when unclear, the area around the ducts within 2 high-power fields. Detailed guidance is available in Supplementary Table 1. Figure 1 shows hematoxin and eosin (H&E) images of TILs density in DCIS. Due to the limited extent (< 1 mm) of the lesions in the DCIS-Mi micro-invasive area, the TILs evaluation guidelines of the International TILs Working Group 2014 [11] are not applicable. We evaluated the DCIS area that was 1 mm away from the infiltrating lesion. Two pathologists evaluated the TILs of 485 specimens sections stained with H&E and reached a consensus.

### Statistical analysis

Statistical analysis uses IBM SPSS version 26.We employed receiver operating characteristic (ROC) curve analysis to determine cutoff values for TILs (in the DCIS and DCIS-Mi groups). TILs are evaluated as a continuous variable, divided into three groups: low (≤ 5%), medium (5–40%) and high (≥ 40%), and use Chi-square test or Fisher's exact test to assess the relationship between TILs and clinicopathological characteristics. Ranked data (tumor nuclear grade) uses the Kruskal Wallis rank sum test. We used the Kaplan–Meier curve to analyze the DFS and the log-rank test for comparison. Univariate, multivariate cox regression model and 95% confidence interval (CI) were used to analyze the significance of the influence of TILs on DFS in BC patients. All tests were two-sided, *P* value < 0.05 was considered statistically significant.

## Results

### Patient description and follow-up

A total of 385 patients were enrolled in this study, of which 360 were DCIS and 125 were DCIS-Mi. The patients were all female, aged 27–76 years (median 48 years).The diameter of the DCIS tumor is 0.3–10.5 cm (median 1.8 cm). The diameter of the DCIS-Mi tumor is 0.5–9 cm (median 3 cm). 90.8% of DCIS patients underwent breast-conserving surgery (BCS) or mastectomy, and 54.1% of DCIS-Mi patients underwent mastectomy. Almost all patients underwent intraoperative sentinel lymph node biopsy (SLNB), postoperative radiotherapy, and corresponding endocrine therapy based on the expression of HR.

The median follow-up time was 42 months (range 4–70 months). 13 cases of DCIS progressed (including 1 of ipsilateral DCIS; 5 of ipsilateral IBC; 5 of contralateral DCIS; 2 of contralateral IBC). 6 cases of DCIS-Mi progressed (including 3 of ipsilateral DCIS; 1 of ipsilateral IBC; 1 of contralateral DCIS; 1 of distant liver metastasis). Of the total 19 end-point events, 13 cases were HER2 overexpression. Details of recurrence patients are in Supplementary Table 2. At the end of the follow-up, no deaths occurred.

### TILs in DCIS and DCIS-Mi patients

Tables 1 and 2 show the relationship between TILs and clinicopathological characteristics, respectively, in DCIS and DCIS-Mi patients. In both groups, larger tumor size, higher nuclear grade, HR negativity are all related to high TILs (*P* < 0.05). High TILs are also associated with high Ki-67 proliferation index in DCIS patients (*P* < 0.001). Age (*P* = 0.815), Ki67 expression (*P* = 0.116) and SLNB status (*P* = 0.415) were not related to the TILs of patients with DCIS-Mi. Obviously, high TILs was significantly correlated with HER2 overexpression (*P* < 0.001). In the TILS ≥ 40% group, 83.7% of DCIS had HER2+ expression (HR + HER2+:25.6%; HR-HER2+:58.1%); 80% of DCIS-Mi HER2+ expression (HR + HER2+: 23.3%; HR-HER2+: 56.5%). TNBC accounts for 11.2% of DCIS-Mi higher than that of DCIS (2.5%).

**Table 1:** Associations between TILs and clinicopathological factors in DCIS BC patients

	All patients patients	TILs			*P* value
		≤ 5%	5–40%	≥ 40%	
	(*N* = 360)	(*N* = 142, 39.4%)	(*N* = 175, 48.6%)	(*N* = 43, 12%)	
*Age*					
< 50 years	181 (50.3)	73 (51.4)	89 (50.9)	19 (44.2)	0.639
≥ 50 years	179 (49.7)	69 (48.6)	86 (49.1)	24 (55.9)	
*Tumor size*					
< 2 cm	186 (51.7)	86 (60.6)	82 (46.9)	18 (41.9)	**0.020**
≥ 2 cm	174 (48.3)	56 (39.4)	93 (53.1)	25 (58.1)	
*Grade*					
Low (G1)	75 (20.8)	48 (33.8)	26 (14.9)	1 (2.3)	** < 0.001**
Intermediate (G2)	123 (34.2)	57 (40.1)	61 (34.9)	5 (11.6)	
High (G3)	162 (45)	37 (26.1)	88 (50.2)	37 (86.1)	
*ER*					
Negative	103 (28.6)	16 (11.3)	58 (33.1)	29 (67.4)	** < 0.001**
Positive	257 (71.4)	126 (88.7)	117 (66.9)	14 (32.6)	
*PR*					
Negative	120 (33.3)	22 (15.5)	67 (38.3)	31 (72.1)	** < 0.001**
Positive	240 (66.7)	120 (84.5)	108 (61.7)	12 (27.9)	
*HER2*					
Negative	156 (43.3)	86 (60.6)	63 (36)	7 (16.3)	** < 0.001**
Positive	204 (56.7)	56 (39.4)	112(64)	36 (83.7)	
*Ki-67*					
≤ 20%	270 (75)	120 (84.5)	127 (72.6)	23 (53.5)	** < 0.001**
> 20%	90 (25)	22 (15.5)	48 (27.4)	20 (46.5)	
*Subtype*					
HR + HER2 −	147 (40.8)	84 (59.1)	59 (33.7)	4 (9.3)	** < 0.001**
HR + HER2+	115 (32.0)	42 (29.6)	62 (35.4)	11 (25.6)	
HR− HER2+	89 (24.7)	14 (9.9)	50 (28.6)	25 (58.1)	
TNBC	9 (2.5)	2 (1.4)	4 (2.3)	3 (7.0)	
*Type of surgery*					
BCS/+ BR	159 (44.1)	77 (54.2)	67 (38.3)	15 (34.9)	**0.038**
Mastectomy	168 (46.7)	55 (38.8)	89 (50.9)	24 (55.8)	
Mastectomy + ALND	33 (9.2)	10 (7.0)	19 (10.8)	4 (9.3)	

Bold indicates p < 0.05

*BCS* breast-conserving surgery, *BR* breast reconstruction, *ALND* axillary lymph node dissection

**Table 2:** Associations between TILs and clinicopathological factors in DCIS-Mi BC patients

	All patients	TILs			*P* value
		≤ 5%	5–40%	≥ 40%	
	(*N* = 125)	(*N* = 29, 23.2%)	(*N* = 66, 52.8%)	(*N* = 30, 24%)	
*Age*					
< 50	67 (53.6)	17 (58.6)	34 (51.5)	16 (53.3)	0.815
≥ 50	58 (46.4)	12 (41.4)	32 (48.5)	14 (46.7)	
*Tumor size*					
< 3 cm	48 (38.4)	15 (51.7)	27 (40.9)	6 (20)	**0.036**
≥ 3 cm	77 (61.6)	14 (48.3)	39 (59.1)	24 (80)	
*Grade*					
Low (G1)	7 (5.6)	2 (6.9)	5 (7.6)	0 (0)	**0.015**
Intermediate (G2)	31 (24.8)	14 (48.3)	11 (16.7)	6 (20)	
High (G3)	87 (69.6)	13 (44.8)	50 (75.7)	24 (80)	
*ER*					
Negative	61 (48.8)	3 (10.3)	38 (57.6)	20 (66.7)	** < 0.001**
Positive	64 (51.2)	26 (89.7)	28 (42.4)	10 (33.3)	
*PR*					
Negative	70 (56)	8 (27.6)	39 (59.1)	23 (76.7)	**0.001**
Positive	55 (44)	21 (72.4)	27 (40.9)	7 (23.3)	
*HER2*					
Negative	50 (40)	21 (72.4)	23 (34.8)	6 (20)	** < 0.001**
Positive	75 (60)	8 (27.6)	43 (65.2)	24 (80)	
*Ki-67*					
≤ 20%	62 (49.6)	19 (65.5)	28 (42.4)	15 (50)	0.116
> 20%	63 (50.4)	10 (34.5)	38 (57.6)	15 (50)	
*Subtype*					
HR+ HER2−	36 (28.8)	19 (65.5)	13 (19.7)	4 (13.3)	** < 0.001**
HR + HER2+	30 (24)	7 (24.1)	16 (24.2)	7 (23.3)	
HR− HER2+	45(36)	1 (3.5)	27 (40.9)	17 (56.7)	
TNBC	14 (11.2)	2 (6.9)	10 (15.2)	2 (6.7)	
*Sentinel lymph node*					
Negative	108 (86.4)	26 (89.7)	57 (86.4)	25 (83.3)	0.415
Positive	10 (8)	1 (3.4)	5 (7.6)	4 (13.3)	
Unknown	7 (5.6)	2 (6.9)	4 (6)	1 (3.4)	
*Type of surgery*					
BCS / + BR	20 (16)	4 (13.8)	10 (15.2)	6 (20.0)	0.940
Mastectomy	84 (67.2)	21 (72.4)	44 (66.7)	19 (63.3)	
Mastectomy + ALND	21 (16.8)	4 (13.8)	12 (18.1)	5 (16.7)	

Bold indicates p < 0.05

*BCS* breast-conserving surgery, *BR* breast reconstruction, *ALND* axillary lymph node dissection

Table 3 compares TILs in DCIS and DCIS-Mi patients. In both groups, medium TILs (5–40%) are dominant (DCIS: 48.6%; DCIS-Mi: 53.8%). In addition, compared with DCIS, DCIS-Mi patients were significantly associated with high TILS (*P* < 0.001). The proportion of high TILS (≥ 40%) group is much higher in DCIS-Mi patients than in DCIS patients (DCIS-Mi vs DCIS: 24% vs 12%, *P* < 0.001).

**Table 3:** Comparison of TILs in DCIS and DCIS-Mi BC patients

	TILs			
	≤ 5% (*N* = 171)	5–40% (*N* = 241)	≥ 40%(*N* = 73)	*P* value
DCIS(*N* = 360)	142 (39.4)	175 (48.6)	43 (12.0)	** < 0.001**
DCIS-Mi(*N* = 125)	29 (23.2)	66 (52.8)	30 (24.0)	

Bold indicates p < 0.05

### TILs in HER2+ patients

Our analysis found that high TILS is not only significantly different between DCIS and DCIS-Mi patients, but also closely related to HER2 expression. Based on this, we selected 279 (204 of DCIS; 75 of DCIS-Mi) cases of HER2+ BC patients for further analysis (Table 4). We found that in the HER2+ group, DCIS-Mi patients were younger than DCIS, but the difference was not statistically significant (*P* = 0.218).It is valuable that DCIS-Mi patients are associated with larger tumor size (*P* < 0.001), HR negativity (*P* = 0.015), higher Ki-67 index (*P* = 0.003) and higher TILs density (*P* = 0.002).There are also differences in surgical options between the two groups of BC patients. DCIS-Mi patients have more axillary lymph node dissection (ALND) (*P* < 0.001), which is affected by sentinel lymph node metastasis.

**Table 4:** Comparison of TILs in HER2+ BC patients (DCIS vs DCIS-Mi)

	All patients (*N* = 279)	DCIS (*N* = 204)	DCIS-Mi (*N* = 75)	*P* value
	(*N* = 279)	(*N* = 204, 73.1%)	(*N* = 75, 26.9%)	
*Age*				
< 50	143 (51.3)	100 (49.0)	43 (57.3)	0.218
≥ 50	136 (48.7)	104 (51.0)	32 (42.7)	
*Tumor size*				
< 2.5 cm	123 (44.1)	110 (53.9)	13 (17.3)	** < 0.001**
≥ 2.5 cm	156 (55.9)	94 (46.1)	62 (82.7)	
*Grade*				
Low (G1)	13 (4.7)	12 (5.9)	1 (1.3)	0.073
Intermediate (G2)	73 (26.1)	58 (28.4)	15 (20.0)	
High (G3)	193 (69.2)	134 (65.7)	59 (78.7)	
*HR*				
Negative	134 (48.0)	89 (43.6)	45 (60.0)	**0.015**
Positive	145 (52.0)	115 (56.4)	30 (40.0)	
*Ki-67*				
≤ 20%	166 (59.5)	132 (64.7)	34 (45.3)	**0.003**
> 20%	113 (40.5)	72 (35.3)	41 (54.7)	
*TILs*				
≤ 5%	64 (22.9)	56 (27.5)	8 (10.7)	**0.002**
5–40%	155 (55.6)	112 (54.9)	43 (57.3)	
≥ 40%	60 (21.5)	36 (17.6)	24 (32.0)	
*Type of surgery*				
BCS/+BR	95 (34.1)	83 (40.7)	12 (16.0)	** < 0.001**
Mastectomy	151 (54.1)	102 (50.0)	49 (65.3)	
Mastectomy + ALND	33 (11.8)	19 (9.3)	14 (18.7)	

Bold indicates p < 0.05

*BCS* breast-conserving surgery, *BR* breast reconstruction, *ALND* axillary lymph node dissection

### TILs and DFS

**Fig. 2:**
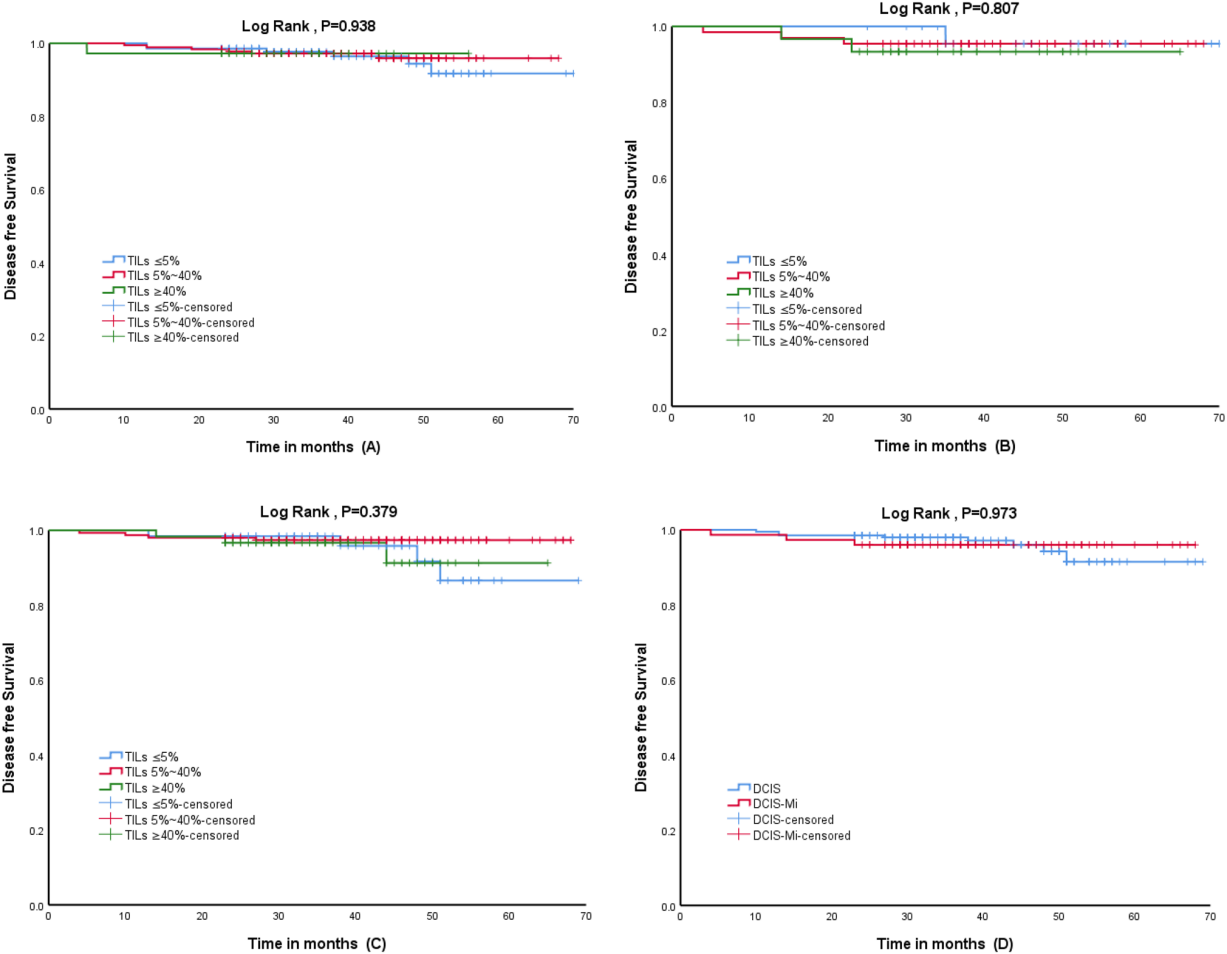
Kaplan–Meier (K-M) curve of DFS in BC patients: **A**: K-M curve of TILs in DCIS patients (*P* = 0.938); **B**: K-M curve of TILs in DCIS-Mi patients (*P* = 0.807); **C**: K-M curve of TILs in HER2+ patients (*P* = 0.379); **D**: K-M curve of DCIS and DCIS-Mi in HER2+ patients (*P* = 0.973)

In the survival analysis, we found that TILs has no prognostic value for DCIS (*P* = 0.938), DCIS-Mi (*P* = 0.807) and HER2+ (*P* = 0.379) BC patients. In addition, there is no difference in the prognosis of BC patients whether DCIS patients are accompanied by microinvasion (*P* = 0.973). The Kaplan–Meier survival curve is shown in Fig. 2.

In the univariate and multivariate cox regression analysis of DFS of BC patients (Table 5), the patients were stratified according to the percentage of TILs (≤ 5%; 5–40% and ≥ 40%). The effect of TILs association on the hazard of DFS adjusted by age, tumor size, histologic grade, HR status, Ki-67 proliferation index, presence of microinfiltrating lesions and type of surgery was negligible in the cox regression model. We confirmed that regardless of DCIS, DCIS-Mi or HER2+ BC patients, no statistically significant correlation between TILs and DFS was observed (in HER2+ group: HR 0.908 comparing ≤ 5% vs. 5–40%, 95% CI 0.196–4.210; and 1.765 comparing ≤ 5% vs. ≥ 40%, 95% CI, 0.309–10.077).

**Table 5:** Univariate and multivariate cox regression model for DFS in DCIS/DCIS-Mi/HER2+ BC patients

TILS	DCIS		DCIS-Mi		HER2+	
	HR (95% CI)	*P*	HR (95% CI)	*P*	HR (95% CI)	*P*
*Univariate analysis*						
≤ 5%	Ref		Ref		Ref	
5–40%	0.816 (0.262–2.543)	0.726	1.342 (0.140–12.902)	0.799	0.443 (0.110–1.775)	0.250
≥ 40%	0.970 (0.114–8.242)	0.978	2.086 (0.189–23.189)	0.549	1.031 (0.227–4.679)	0.969
Multivariate cox regression model*						
≤ 5%	Ref		Ref		Ref	
5–40%	0.792 (0.244–2.569)	0.697	2.075 (0.168–25.666)	0.570	0.908 (0.196–4.210)	0.902
≥ 40%	0.813 (0.082–8.084)	0.860	4.295 (0.184–100.316)	0.365	1.765 (0.309–10.077)	0.523

*Hazard ratios (HR) adjusted by age, tumor size, histologic grade, HR status, Ki-67 proliferation index, presence of microinfiltrating lesions and type of surgery in a cox proportional hazard model

## Discussion

In our study of Chinese population, TILs are all related to some poor prognostic clinicopathological characteristics (such as larger tumor size, HR negativity, higher nuclear grade) both in DCIS patients and DCIS-Mi BC patients. Some studies have found racial differences in the pathological characteristics and treatment modalities of DCIS patients [12, 13]. Moreover, DCIS-Mi patients are associated with higher TILs than DCIS patients (*P* < 0.001). Some previous studies [5, 14–16] have also obtained similar results. With tumor infiltration, the density of TILs tends to increase. In DCIS, the most common tumor is HR + /HER2- subtype (40.8%, *P* < 0.001). However, in the DCIS-Mi group, the proportion of HR-/HER2+ tumors was the highest (36%, *P* < 0.001), and the TNBC subtype was more common than in DCIS. Kim et al. reached similar results [17]. Interestingly, among DCIS-Mi patients, HR-/HER2+ tumors accounted for a rather alarming proportion (56.7%) in the high TILs (≥ 40%) group. This result confirms that HER2 overexpressing tumors are quite immunogenic [14], which can be reflected by the density of TILs. Therefore, we selectively analyzed all HER2+ patients and found that compared with HER2+ DCIS patients, HER2+ DCIS-Mi patients had larger tumors (*P* < 0.001), HR negative (*P* = 0.015), and higher Ki-67 expression (*P* = 0.003) and high TILs (*P* = 0.002). This indicates that in the process of HER2+ DCIS invasion, TILs have undergone a series of changes with the change of TME, and one of the changes has increased the density of TILs.

In addition, we found that microinfiltration has no effect on the prognosis of DCIS (*P* = 0.973), even though DCIS-Mi tumors seem to have worse biological behaviors than DCIS. A large study based on the Surveillance, Epidemiology and End Results (SEER) registries database showed that compared with DCIS patients, microinvasive carcinoma have an increased risk of BC death, and their prognosis more closely resembles small invasive cancer (0.2–1.0 cm) [18]. The current treatment model and prognosis are comparable to those of small-volume invasive cancer [19]. In addition, de Boniface et al. [20] confirmed that BCS with postoperative radiotherapy is better than mastectomy without radiotherapy for the survival of DCIS patients.

The survival analysis showed that TILs has no correlation with DFS in DCIS (*P* = 0.938), DCIS-Mi (*P* = 0.807) and HER2+ (*P* = 0.379) BC patients. Unfortunately, the short follow-up time was a shortcoming of our study, and TILs had no effect on short-term DFS in BC patients. We used the same stromal TILs assessment guidelines as Pruneri et al. [5]. They studied 1488 patients with DCIS and found no significant association between TILs and the risk of 10-year ipsilateral breast event (IBE). According to the different distribution of TILs in DCIS, the current assessment methods can be roughly divided into stromal TILs, hotspot-TILs, and touching-TILs. After comparing seven TILs evaluation methods, Toss et al. [16] found that touching-TILs had the strongest correlation with the results. Xu et al. [21] used the above-mentioned touching-TILs assessment method, which was defined by TILs touching or within one lymphocyte cell thickness from the malignant ducts’ basement membrane. Similarly, they confirmed that "Touching-TILs" > 5 is an independent prognostic factor for higher ipsilateral breast tumor recurrence (IBTR). Based on different TILs evaluation criteria, many studies [22, 23] have confirmed that high-density TILs is associated with poor prognostic parameters and predicting recurrence of DCIS. Moreover, the cut-off values for the risk stratification of TILs in the current study are quite different. We believe that these factors may be one of the reasons that cause our prognostic significance to be different from other related studies. As far as we know, our study is one of the few to evaluate stromal TILs according to the guidelines recommended by the International Immuno-Oncology Biomarkers Working Group. In order to deeply study the role of TILs in the process of tumor invasion, a unified and easily quantified evaluation standard is essential. From the current research, touching-TILs seems to have more potential research value.

For a long time, BC has not been regarded as a typical immunogenic tumor [24]. In the TME, tumor cells interact with infiltrating immune cells, causing immunosuppression to promote immune escape of tumor cells. The immune evasion mechanism of BC is not yet clear [25]. During the invasion of DCIS, the changes of TILs may be complicated. In addition to the analysis of the density of TILs, some studies [2, 26–28] have carried out studies on the changes of TILs subsets and immune checkpoint proteins expression during this process, trying to explore the role and predictive significance of specific lymphocytes in the invasion process. Programmed cell death-1 (PD-1) receptors are up-regulated on activated T cells and interact with programmed cell death ligand 1 (PD-L1) on the surface of tumor cells and immune cells to produce immunosuppression to weaken the body's anti-tumor effect [29]. Kim et al. [26] found that in DCIS, the high infiltration of CD4 +, CD8 + and FOXP3 + T cells and the presence of PD-L1 + immune cells were related to clinicopathological features of worse biological behavior, such as high nuclear grade, comedo-type necrosis, and high Ki-67 proliferation index. Moreover, high infiltration of FOXP3 + TILs and the presence of PD-L1 + immune cells were associated with tumor recurrence in DCIS patients. Thike et al. [22] found that both CD4 + T cell density and CD4/CD8 ratio were related to recurrence and ipsilateral invasive recurrence. In summary, although it is difficult to uniformly quantify the evaluation of TILs subsets in IHC slices, several studies have proved its potential predictive and prognostic value [2, 30]. PD-L1 is currently the most effective predictive biomarker for cancer immunotherapy. In addition, many scholars also recognize PD-L1 as a potential marker for DCIS disease progression and recurrence [28]. Since TILs and PD-L1 are part of the BC immune spectrum, the International Immuno-Oncology Biomarker Working Group recommends the systematic implementation of combined PD-L1 and TILs analysis as a more comprehensive immuno-oncology biomarker for screening BC patients PD-1/ PD-L1 inhibitory treatment [31].

The latest research shows that adoptive cell therapy (ACT) is a promising solid tumor immunotherapy, which uses the patient’s own immune cells to eliminate tumor cells [32]. Importantly, T-lymphocyte-based ACT (TIL ACT) has received great attention. In TIL ACT, TILs are collected from resected tumor tissues, enhanced and expandedex-vivo, and delivered back to the patient as therapeutic agents. In several cancers, including melanoma, cervical squamous cell carcinoma, and cholangiocarcinoma, TIL ACT causes tumor regression [33]. The enrichment of PD-L1 in TME promotes the immune system's tolerance to tumors. The overexpression of PD-L1 has been proven to inhibit the anti-tumor immune response mediated by T cells, leading to tumor evasion of immunity [34]. In BC, due to high levels of tumor PD-L1 are likely to inhibit the activity of transferred TILs within the TME, the efficacy of PD-1/PD-L1 targeted drugs is more limited. Combining TIL ACT with anti-PD-1/PD-L1 therapy is a good solution, and it may make an important contribution to BC immunotherapy [35]. TILs have considerable value in predicting the prognosis of BC and tumor immunotherapy, and a large number of prospective studies are needed to confirm it.

In conclusion, our study found the difference between TILs in DCIS and DCIS-Mi patients in the Chinese population, and focused on analyzing the characteristics of TILs in HER2 overexpressing tumors. The density of TILs tends to increase during tumor invasion. Unfortunately, we have not found direct evidence that TILs are related to the prognosis of DCIS, but we cannot deny the value of ignoring TILs. We will further study the subset of TILs in DCIS and DCIS-Mi to provide more data for DCIS management and BC immunotherapy support.

## Supplementary Information

Below is the link to the electronic supplementary material.

Supplementary file1 (DOC 68 KB)
