# Supplementary material for: Clinicopathological characteristics and prognostic analysis of tumor-infiltrating lymphocytes (TILs) in ductal carcinoma in situ (DCIS) and DCIS with microinvasion (DCIS-Mi) of the breast
Source: Breast Cancer Res Treat. 2022 Mar 8;193(1):111–20. doi: 10.1007/s10549-022-06553-z (PMC8993775; doi:10.1007/s10549-022-06553-z)
Supplement: Supplementary file 1 — Supplementary file1 (DOC 68 KB) [file 10549_2022_6553_MOESM1_ESM.doc]

**Supplementary tables**

| **Supplementary Table 1. TILs assessment in *in situ* (CIS)-lesions of the breast: guidelines from the TILs Working Group.** | |
| --- | --- |
| 1 | TILs can be scored for each type of CIS, whether it is ductal or lobular. TILs surrounding areas of lobular cancerization by a DCIS or ductal pagetoid spread by a LCIS should also be registered. This should be specified in the report and in advance. The same methodology applies to whether it concerns lobular or ductal carcinoma in situ. |
| 2 | TILs can be scored surrounding CIS as part of an invasive tumor or as CIS with no invasive component. |
| 3 | The methodology for scoring TILs surrounding CIS as part of an invasive tumor or as CIS with no invasive component is exactly the same. |
| 4 | If TILs are scored as part of an invasive tumor, CIS at a distance from the edge of the invasive tumor on the same slide should also be taken into account in the final TILS CIS-score. TILs should be reported for the stromal compartment (= % stromal TILs).  The denominator used to determine the % stromal TILs is the area of stromal tissue (i.e. area occupied by mononuclear inflammatory cells over total intratumoral stromal area), not the number of stromal cells (i.e. fraction of total stromal nuclei that represent mononuclear inflammatory cell nuclei).  The stromal compartment is defined as the area within the specialized stroma surrounding the CIS-lesions, or if this specialized stroma is not clearly visible the TILs clearly surrounding the CIS should be assessed. This is defined as the area surrounding the duct within two high-power microscopic fields.  If many CIS lesions are encountered, an evaluation of the area surrounding all lesions should be assessed, and the % stromal TILs in the total stromal area of all CIS-lesions should be used.  Any type of circumferential TILs-infiltration is taken into account, thus also a minimal, partial, subtotal and total circumferential TILs-infiltration around CIS-lesions.  TILs that are in continuity between the invasive lesions and the in situ lesions with no clear distinction as to whether these are TILs associated with the invasive or CIS-component are not considered to be part of the to be assessed CIS TIL-component.  There should be no distinction to be made between TILs surrounding CIS-lesions of different differentiation grades. |
| 5 | TILs located within CIS-lesions, in between the *in situ* tumor cells, should not be assessed. |
| 6 | Exclude TILs around normal lobules, wherever they are found. |
| 7 | Exclude TILs in tumor zones with crush artifacts, necrosis, regressive hyalinization as well as in the previous core biopsy site. |
| 8 | All mononuclear cells (including lymphocytes and plasma cells) should be scored, but polymorphonuclear leukocytes are excluded. |
| 9 | An evaluation of all sections containing CIS is recommended (4-5 µm, magnification 200-400x) per patient. Full sections are preferred over biopsies, whenever available. |
| 10 | Cores or vacuum assisted biopsies can be used in the pre-therapeutic neoadjuvant setting; currently no validated methodology has been developed to score CIS TILs after neo-adjuvant treatment. |
| 11 | A full assessment of average TILs in the CIS-tumor area by the pathologist should be used. Do not focus on hotspots. |
| 12 | TILs may provide more biological relevant information when scored as a continuous variable, since this will allow more accurate statistical analyses, which can later be categorized around different thresholds. However, in daily practice most pathologists will rarely report for example 13.5% and will round up to the nearest 5-10%, in this example thus 15%. Pathologist should report their scores in as much detail as the pathologist feels comfortable with. |
| 13 | TILs should be assessed as a continuous parameter. The percentage of stromal CIS-TILs is a semiquantitative parameter for this assessment, for example, 80% stromal CIS-TILs means that 80% of the stromal area shows a dense mononuclear infiltrate. For assessment of percentage values, the dissociated growth pattern of lymphocytes needs to be taken into account. Lymphocytes typically do not form solid cellular aggregates, therefore the designation “100% stromal CIS-TILs” would still allow some empty tissue space between the individual lymphocytes. |
| 14 | No formal recommendation for a clinically relevant CIS TIL threshold(s) can be given at this stage. |

| **Supplementary table2:Clinicopathological characteristics of recurrent BC patients** | **Outcome** | Ipsilateral .DCIS | Ipsilateral .DCIS | contralateral .DCIS | Ipsilateral .IBC | Ipsilateral .IBC | Ipsilateral .IBC | Ipsilateral .DCIS | Ipsilateral .IBC | Ipsilateral .DCIS | liver metastases | Ipsilateral .DCIS | contralateral .DCIS | contralateral .IBC | contralateral .DCIS | contralateral .DCIS | contralateral .DCIS | Ipsilateral .IBC | Ipsilateral .IBC | contralateral .IBC | Abbreviations: BR=breast reconstruction; ALND=axillary lymph node dissection |
| --- | --- | --- | --- | --- | --- | --- | --- | --- | --- | --- | --- | --- | --- | --- | --- | --- | --- | --- | --- | --- | --- |
|  | **Survival/m** | 14 | 23 | 44 | 38 | 14 | 27 | 4 | 48 | 10 | 22 | 13 | 35 | 24 | 51 | 13 | 9 | 13 | 29 | 19 |  |
|  | **radiotherapy** | - | - | + | + | - | - | - | + | + | - | - | - | + | + | + | + | - | - | - |  |
|  | **Endocrine therapy** | - | - | - | + | + | + | - | + | + | + | - | + | + | + | + | - | + | - | + |  |
|  | **Subtype** | Mastectomy | Mastectomy | Lumpectomy+BR | Lumpectomy+BR | Mastectomy+ ALND | Mastectomy | Mastectomy | Lumpectomy+BR | Lumpectomy | Mastectomy | Mastectomy | Mastectomy | Lumpectomy+BR | Lumpectomy | Lumpectomy | Lumpectomy | Mastectomy+ ALND | Mastectomy | Mastectomy |  |
|  | **Sentinel lymph** | - | - | - | - | + | - | - | - | - | - | - | - | - | - | - | - | + | - | - |  |
|  | **Microinvasive** | + | + | - | - | + | - | + | - | - | + | - | + | - | - | - | - | - | - | - |  |
|  | **HER2** | - | + | + | + | + | + | + | + | + | - | + | + | - | + | + | - | - | + | - |  |
|  | **HR** | - | - | - | + | + | + | - | + | + | + | - | + | + | + | + | + | + | - | + |  |
|  | **TILs/%** | 35 | 40 | 40 | 5 | 50 | 10 | 30 | 5 | 20 | 20 | 10 | 5 | 15 | 5 | 5 | 55 | 5 | 5 | 30 |  |
|  | **Grade** | G3 | G3 | G3 | G2 | G3 | G3 | G3 | G3 | G3 | G3 | G3 | G3 | G2 | G3 | G2 | G3 | G2 | G3 | G1 |  |
|  | **Size/cm** | 9 | 6 | 4 | 2 | 5 | 7 | 7 | 3 | 0.6 | 2.5 | 3 | 1.5 | 1.7 | 1.2 | 0.5 | 0.5 | 2 | 2.5 | 1.5 |  |
|  | **Age/y** | 29 | 32 | 34 | 35 | 36 | 37 | 37 | 41 | 42 | 42 | 43 | 45 | 47 | 47 | 50 | 50 | 51 | 51 | 69 |  |
|  | **No.** | 1 | 2 | 3 | 4 | 5 | 6 | 7 | 8 | 9 | 10 | 11 | 12 | 13 | 14 | 15 | 16 | 17 | 18 | 19 |  |
